# Supplementary figures and images for: Pneumococcal colonisation is an asymptomatic event in healthy adults using an experimental human colonisation model
Source: PLoS One. 2020 Mar 10;15(3):e0229558. doi: 10.1371/journal.pone.0229558 (PMC7064211; doi:10.1371/journal.pone.0229558)

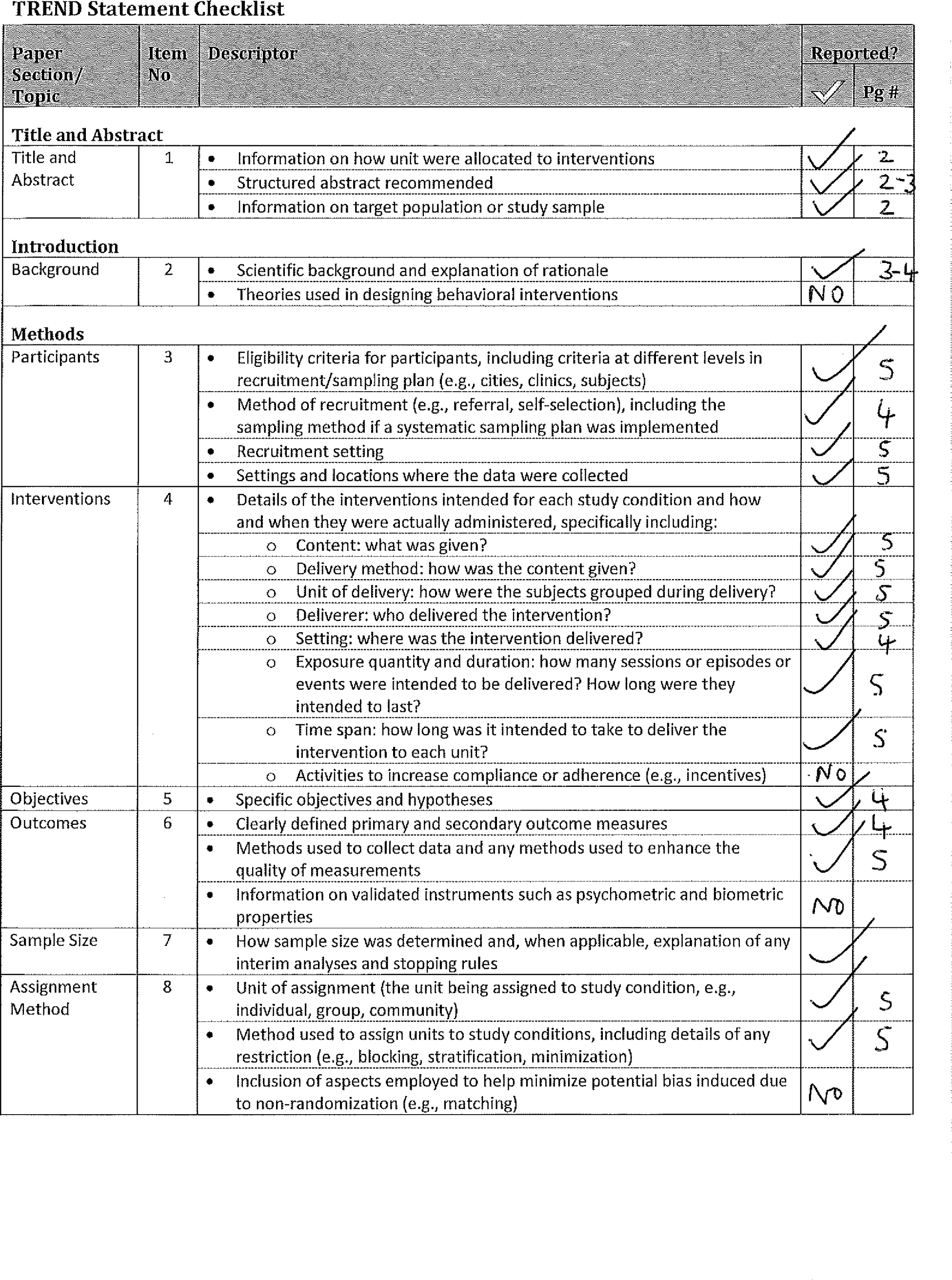

Supplement: S1 Fig — (TIF) [file pone.0229558.s001.tif]
