# Supplementary material for: Pneumococcal colonisation is an asymptomatic event in healthy adults using an experimental human colonisation model
Source: PLoS One. 2020 Mar 10;15(3):e0229558. doi: 10.1371/journal.pone.0229558 (PMC7064211; doi:10.1371/journal.pone.0229558)
Supplement: S2 Fig — (PDF) [file pone.0229558.s002.pdf]

PROTOCOL - Experimental Human Pneumococcal Carriage (EHPC)

Background

Clinical problem

Streptococcus pneumoniae is the leading cause of morbidity and mortality due to community acquired pneumonia (CAP), bacterial meningitis and bacteraemia worldwide [1]. Pneumococcal infections cause over 1 million pneumonia deaths per year in children in the developing world as well and are also a major burden of otitis media globally.

Current vaccines

Capsular polysaccharide vaccination (PPV) provides protection against invasive pneumococcal disease but is less protective against pneumonia in adults [2] and is ineffective in young children. Protein-conjugated pneumococcal polysaccharide (PCV) vaccines represent a landmark achievement showing good serotype-specific immunity against invasive syndromes (83-94%) in children and an important herd immunity effect in adults, but they are not a final solution [3]. Conjugate vaccine (PCV) is difficult to manufacture, expensive, limited in serotype coverage, associated with serotype replacement and is less effective against mucosal infection (13-20%) than against invasive disease [4] so alternative strategies are still urgently needed [5]. This urgency is further increased by a global increase in antibiotic resistance among pneumococci and the number of susceptible people. Pneumococcal disease is a major global health threat for which new vaccines are urgently needed, particularly those that will protect vulnerable children and adults against pneumonia.

The relation of pneumococcal carriage and disease

Pneumococcal disease is preceeded by colonisation of the nasopharynx of uninfected adults and children. ‘Asymptomatic’ colonisation may be followed by carriage which remains asymptomatic or may (rarely) progress to disease. Most episodes of carriage result in improved immunity to future infection without the development of disease. As such, the mechanisms by which carriage is established, the immunity that results from carriage and the mechanisms that determine when carriage ends are all of critical importance in the design of mucosal vaccines against pneumococcal disease.

Immune responses to carriage

Although there are many studies measuring natural pneumococcal carriage rates and serotypes in humans, there are very limited data on the immune response to pneumococcal carriage in humans because the timing of any natural carriage episode is difficult to determine. Experimental human pneumococcal carriage has been safely established in two previous studies.[6, 7] From these studies, it is known that a variable proportion of those subjects inoculated with pneumococci become colonised. The variables determining carriage/non-carriage following inoculation are unclear. The rate of colonisation following nasal inoculation in these studies was reported as <50%, with a duration of carriage between 21 and 122 days. The immune response in nasal washings and serum showed an association between humoral responses (to protein and polysaccharide antigens) and carriage. The humoral and cellular responses in the lung that result in response to nasopharyngeal pneumococcal carriage have not been described.

Pneumococcal antigens and humoral response

Murine studies have identified a number of pneumococcal proteins on the cell surface that induce antibodies in the host including pneumococcal surface protein A (PspA), pneumococcal surface adhesion A (PsaA) and choline binding protein A (CbpA, also called PspC). These proteins are common to the cell surface of all pneumococci. In addition, pneumolysin (Ply) is a cytoplasmic protein also expressed by all pneumococci. These surface and cytoplasmic proteins are both highly immunogenic and humoral responses confer protection against both invasive disease and carriage in mice [8-12]. Pre-existing antibody to cell wall polysaccharide has been demonstrated to be predictive of resistance to colonisation [6, 7]. Susceptibility to carriage does not appear

to correlate with pre-existing levels of immunoglobulin G (IgG) to the specific capsular polysaccharide. A rise in specific antibodies to protein (CbpA and PspA) during human carriage has also been demonstrated [6, 7].

Mucosal cellular responses to pneumococci

It has been demonstrated that mice lacking effective T-cell function have an increased duration of pneumococcal colonisation following inoculation, suggesting that further examination of the cellular immune response to colonisation may provide useful information [13]. Mucosal T lymphocytes are critical in the regulation of mucosal defence. Recent data have emphasized the importance of both humoral responses and Th17 subsets of CD4 lymphocytes in mucosal defence in murine model [14, 15]. Our group has recently demonstated the up-regulation of Th17 lymphocytes in human lung following EHPC. Up-regulation of Th17 antigen-specific cells is consistent with enhanced protection against pneumonia; this could be tested in future by observing the association between Th17 response and vaccine protection against a pneumococcal challenge.

Important current questions

This study will address important current questions including dose dependency to establish pneumococcal carriage in human subjects, the immune response in the lower airway to nasal carriage and innate, humoral and cellular responses in both mucosal and systemic compartments. We have previously demonstrated these responses to polysaccharide and conjugate vaccines [16-18]. Examination of individuals pre and post pneumococcal carriage, and comparison between the immune response of colonised against un-colonised subjects will provide new information on the innate, cellular and humoral immune responses to pneumococcal colonisation. In future this model will be used in testing the effect of new candidate vaccines. A major roadblock identified in the process of developing new protein vaccines has been a means of down-selecting from among the candidate antigen combinations proposed as vaccines - the EHPC model offers a means of doing just this by testing the effectiveness of candidate vaccines in blocking subsequent EHPC.

Study design overview

We will carry out (a) **dose-ranging** study using 10 experimental carriage volunteers at each of 6 test doses and construct a dose-response curve, (b) prospectively test the **reproducibility** and confidence interval associated with 50-80% carriage using Lot Quality Assurance Sampling (LQAS), (c) **vaccine effect testing**.

- **Dose-ranging study** – Healthy volunteer adults will be screened to exclude subjects at risk of disease or subjects in contact with others at risk. Recruited volunteers will be screened for native pneumococcal carriage by nasal wash. Natural carriage volunteers will be excluded from inoculation but continue with the rest of the study as per experimental carriage volunteers. 120 experimental carriage subjects will then be recuited in groups of 10 subjects. Up to 40 natural carriage volunteers will be recruited. Each group of 10 experimental carriage subjects will be inoculated with 0.1ml pneumococci to each nostril. The inoculated doses will be increased in each cohort of ten subjects from the initial cohort ($1x10^4$ cfu/nostril), to $2x10^4$, $4x10^4$, $8x10^4$, $1.6x10^5$, $3.2x10^5$ cfu/nostril. We plan to recruit up to 20 participants to receive an inoculation of 0.9% saline solution to provide a control arm. We will use the data to construct a graph of the carriage rate achieved with each inoculum dose. The full dose ranging study will be done with serotype 6B and the repeated with serotype 23F – both of these types have been used in our pilot studies.

- The **primary endpoint** in each subject will be the detection of pneumococci by classical culture methods in nasal wash at 48hrs and/or 7-10 days following inoculation. The dose-response curve will then be used to tailor future vaccine assessment studies to purpose. Predicted 80% carriage will allow the EHPC model to have high sensitivity for vaccine efficacy with small study numbers. The dose-response curve will also allow prediction of a 50% endpoint that will allow the combined testing for benefit and harm in future vaccine studies, but at the cost of larger study numbers.

- The **secondary endpoints** are immunological assays to determine defence against infection. We will collect samples in order to apply these assays to nasal wash, saliva, bronchoalveolar lavage, urine, throat

swabs and blood before and after experimental challenge. This will provide data on the density of colonisation as well as upper and lower airway innate, humoral and cellular defence. These data will allow us to define the host variables that predict EHPC reproducibility. For example, it may be that subjects with pre-existing high levels of anti-capsular IgG or innate factors are resistant to EHPC and a future highly reproducible model may have to exclude these subjects at screening.

- **Reproducibility study** - We will recruit 30 experimental carriage volunteers for each of the 2 serotypes. A target carriage rate will be chosen from the dose ranging study and using the dose and protocol refined in that study, simple reproducibility will be assessed by obtaining a confidence interval for the mean carriage. Using more complex data (carriage density, duration) LQAS [19] analysis can be applied. LQAS allows each sample to be defined as acceptable or unacceptable within a range of values and the hypothesis that the ratio of acceptable:unacceptable is the same can be tested in the most parsimonious manner, together with an estimate of the predictive value of this estimate. LQAS combines modelling with hypothesis testing and is directly applicable to the vaccine down-selection planned later.

- We will **re-challenge** volunteers successfully colonized in the reproducibility study with either the homologous or heterologous serotype using the same protocol (re-challenge protocol) to determine within serotype and across-serotype protection. This is optional to the volunteer.

- We will inoculate natural carriers that were recruited during the study with the 6B strain to determine within serotype and across-serotype protection accounting for the previous natural serotype. This will be performed according to the re-challenge protocol however this will include those volunteers that were previously removed from the study due to natural carriage. The 2-6month interval will be counted from the termination of study in those that were previously removed from the study due to natural carriage or from completion of study in all other natural carriers. This is optional to the volunteer.

Aims

The study aims to investigate the innate, acquired humoral and cellular immune response to induced pneumococcal nasal carriage in a safe and controlled manner. We will carry out a dose-ranging study with type 6B and 23F pneumococci in order to find the optimal dose for 50-80% carriage with each serotype (n=10 experimental carriage subjects per dose range). We will then test the hypothesis that we have achieved 50-80% carriage using Lot Quality Assurance Statistics in 2 prospective test sets (n=30 experimental carriage subjects per test set) to ensure reproducibility. We will re-challenge carriers with pneumococci to determine the protective effect of experimental human pneumococcal carriage (EHPC) itself as a surrogate for vaccine induced immunity. We will describe the mechanisms underlying EHPC +/- protection from carriage.

Three major applications of this study are (a) as an immunological probe of mucosal immunity in the upper and lower airway, (b) as a mucosal vaccine model and (c) as a potential surrogate of protection for testing novel protein vaccines (with the eventual goal of a needle-free inhaled vaccine against *S. pneumoniae*).

Subjects and timelines

We will inoculate healthy non-smoking adult volunteers with a well-characterised penicillin-sensitive pneumococcus (23F or 6B) and observe the volunteers for the development of pneumococcal carriage. Colonisation may be expected to occur in 50-80% of the group while the remainder will clear the inoculum in a few hours.

Outline timetable of **dose-ranging study** for volunteers:

- Week 0 Initial appointment to discuss potential study involvement

- Week 1 Appointment for consent, initial nasal wash, urine, throat swab, saliva and blood (serum) collection

- Week 2 If no natural carriage found from initial nasal wash - 0.1ml of either 23F or 6B is inoculated into each nostril. Daily contact with researcher for 7/7 by phone ensues
- Week 2 Nasal wash (48hrs post inoculation), throat swab and urine sample
- Week 3 Nasal wash (7 days post inoculation)
- Week 4 Nasal wash, saliva, blood and urine collection. Amoxicillin 500mg tds for 3 days in all volunteers with carriage.
- Week 10-20 Blood sample

Outline timetable of **reproducibility study** for volunteers:

- Week 0 Initial appointment to discuss potential study involvement
- Week 1 Appointment for consent, initial nasal wash, saliva, blood, throat swab and urine collection
- Week 2 If no natural carriage found from initial nasal wash - 0.1ml of either 23F or 6B is inoculated into each nostril. Daily contact with researcher for 7/7 by phone ensues
- Week 2 Nasal wash (48hrs post inoculation), throat swab and urine
- Week 3 Nasal wash, blood and urine (7 days post inoculation)
- Week 4 Nasal wash, blood and urine
- Week 5 Nasal wash, blood and urine
- Week 6 Nasal wash, blood and urine. Amoxicillin 500mg for 3 days in all volunteers with carriage
- Week 7-9 Nasal wash, saliva, blood and urine collection. Bronchoscopy for BAL collection (when consent obtained as per separate consent form attached)
- Week 10-20 Blood sample

Outline time-table for re-challenge study; a**fter a 2-6 mth time interval of completing the study volunteers are re-challenged with a timeline as follows (this is optional for the volunteer):**

- Week 0 Initial appointment to discuss potential study involvement
- Week 1 Appointment for consent, initial nasal washand blood collection
- Week 2 If no natural carriage found from initial nasal wash - 0.1ml of either 23F or 6B is inoculated into each nostril. Daily contact with researcher for 7/7 by phone ensues
- Week 2 Nasal wash(48hrs post inoculation)
- Week 3 Nasal wash (7 days post inoculation)
- Week 4 Final sample collection (nasal wash and blood).

Amoxicillin 500mg for 3 days in all volunteers with carriage at any time point.

Enriched sample set collection for secondary endpoints:

a) In a sub-set of subjects, consent may be obtained to collect venous blood on days 2, 3, 4, 5 and 10 in addition to the samples planned on days 0, 7, 14, 21 and weekly to week 7 as detailed above. Subjects in the reproducibility study will all be invited to take part in this limb of the study; a small number of subjects in the dose-ranging study will be invited if carriage is likely to occur (higher inoculum doses).

In subjects willing to undergo bronchoscopy, this will be carried out after the termination of carriage/full protocol using a separate consent form to the simple EHPC consent. Subjects in the reproducibility study will all be invited to take part in this limb of the study; subjects in the dose-ranging study will only be invited if carriage occurs.

Method details

1. Recruitment and selection

Advertisements will be placed on physical notice boards in public areas and on the intranet of Liverpool University and RLBUHT, as well as in the local press inviting healthy volunteers to participate. Interested persons are asked to contact the research team for further information and an initial appointment will be made should they wish to consider participating.

Inclusion criteria:

- Adults aged 18-60 yrs - ages chosen to minimise the risk of pneumococcal infection
- Fluent spoken English - to ensure a comprehensive understanding of the research project and their proposed involvement

Exclusion criteria:

- Contact with at risk individuals (children, immunosuppressed adults, elderly, chronic ill health) - minimise risk of pneumococcal transmission
- Current smoker or significant smoking history (>10 pack yrs) – minimise risk of pneumococcal disease and optional bronchoscopy
- Asthma or respiratory disease – to minimise risk of bronchoscopy (can still partake in study)
- Pregnancy - minimise risk of pneumococcal disease
- Allergic to penicillin
- Involved in another clinical trial unless observational or in follow-up (non-interventional) phase.
- Unable to give fully informed consent

2. Screening and preliminary assessment

- Clinical examination - the initial clinic visit will include a focused clinical history and targeted clinical examination involving auscultation of the lung fields and heart sounds. Should a previously unrecognised abnormality be identified this will be explained to the individual, who will be informed, they will be excluded from enrolling in the study and appropriate follow up and investigation will be arranged through the General Practitioner.

- Saliva samples - will be collected as per timetabled protocol above including the first clinic visit. Samples will be collected using salivates retained for 2 minutes between the gum and buccal mucosa as per the manufacturer's instructions.

- Nasal lavage - will be performed using a modified Naclerio method [1]. This is a well used and validated technique to collect nasal cytology specimens with which we now have 2 years experience. Briefly, 10ml of saline is instilled and held for a few seconds in the nares before being expelled in to a petri dish. In the event of nasal wash loss (defined as cough/sneeze/swallow) the procedure may then be repeated to obtain an adequate specimen. Should this initial wash demonstrate that a potential volunteer is already colonised with pneumococci, they will follow the study but without inoculation.

- Blood – venous blood will be taken by an appropriately trained team member. In the standard visits, up to 40ml of blood will be collected for extraction of serum and whole blood, an EDTA sample will be taken on the screening visit to determine normal range of white cell count. In the enriched sample sets, 2 Paxgene tubes (each 5ml) will be collected on the additional days but the 40ml draw will only occur on the standard days. Volunteers will therefore give 40ml per week normally, or 80ml in week 1 and 50 ml in week 2 in the enriched sampling.

- A viral throat swab will be obtained to assess viral load and viral co-infections in individuals who are colonised and not colonised following inoculation. Samples will be taken pre and 48 hours post pneumococcal inoculum to investigate the relationship between upper respiratory tract viral infections and susceptibility and density of pneumococcal colonisation. In brief, the individuals tongue will be depressed using a tongue depressor exposing the palatopharyngeal arch. A sample will be obtained by making 5 small circular motions of the right palatopharyngeal in contact with the mucosa whilst avoiding the patients tongue. The swab will be returned to the respective container and viral multiplex PCR will be used to evaluate the presence of Flu A, Flu B, RSV, hMPV, Paraflus 1-4, adenovirus, rhinovirus, and coronaviruses OC43, 229E, NL63, and HKU1.

3. Preparation of bacterial stock

- **Preparation of bacteria for carriage studies** - mid-log broth culture of pneumococcus (type 23F or 6B) will be frozen at -80°C in aliquots of glycerol-enriched media. Frozen aliquots will be thawed and checked for cfu/ml, E-test penicillin susceptibility and purity. These checks will first be carried out in our laboratory and then identification and characterisation will be confirmed in a reference laboratory. On experimental days, aliquots will be thawed, washed twice, and re-suspended at an appropriate density for each inoculation dose.

- **Inoculation** - using a P200 micropipette 0.1ml broth containing the desired dose (ranging from $1x10^4$ to $3.2x10^5$ cfu/nostril) of type 23F or 6B pneumococcus will be instilled. Patient will be seated in a semi-recumbent position. After inoculation, patient will remain in this position for up to 15mins. Patient will be given post-inoculation advice sheet (including emergency contact details), thermometer, amoxicillin and a daily symptom log to be completed by the participant.

- **Determination of colonisation** - colonisation will be defined by the result of nasal washes taken at 48hrs and 7 days post inoculation. Nasal washes will be plated on to culture media and incubated overnight at 37°C in 5% CO2. Colonies will be confirmed as S. *pneumoniae* using classical techniques including (i) typical draughtsman-like colony morphology (ii) the presence of α-haemolysis (iii) optochin sensitivity (iv)solubility in bile salts and (v) Gram-positive diplococci. Typing by Quellung reaction will be done using a typing kit with specific sera to confirm inoculated pneumococcal types. Isolates will be frozen at -80ºC for storage and reference laboratory confirmation. Results from the cultured nasal wash will also be confirmed using PCR based methods of bacterial detection.**Monitoring of colonisation** - monitoring of colonisation will be done by weekly nasal washes (reproducibility study). Home monitoring will include a

symptom check-list with a clear flow chart of the necessary intervention should any symptoms develop (see patient information sheet). A three day course of amoxicillin will be issued on enrolment. Participants will be required to make text message contact with a specified member of the research team before 1400hrs every day until study completion. Should they not make contact by the specified time; a member of the research team will contact the volunteer. If no contact is made then a prior defined 'secondary contact' will be telephoned. During the post inoculation period volunteers will have access to a 24/7 on-call telephone service until after the end of the study.

4. Termination of carriage

All study participants who are still carrying pneumococcus at the end of the study, will take amoxicillin 500mg tds for 3 days orally at the end of the study.

5. Response measurements

The study primary outcomes are carriage at 48 hours and/or 7days in inoculated volunteers. This will be determined by culture of the 48 hour +/- 7 day nasal wash sample.

Secondary endpoints are immunological parameters and further bacterial carriage characterisation. In particular, innate, humoral and cellular responses to pneumococcal proteins and polysaccharide measured in serum, saliva, nasal wash.

- **Innate Responses**

- Soluble factors - serum, saliva, nasal wash supernatant and BAL supernatant will be assayed for anti-microbial peptides such as SLPI, lysozyme, lactoferrin, LL-37 and beta defensins as well as neutrophil recruiting chemokines such as CXCL-1, 2 (and their receptors) and CXCL-8, IL-8, SLPI.

- Cellular responses - nasal washings, saliva and BAL will be examined by Giemsa stained cytospin for recruitment of neutrophils. In addition, the number of macrophages and the apoptotic fraction of these macrophages will be determined if possible. NB the cellular fraction of nasal wash is highly variable and many samples may have inadequate cellular material for analysis. It may be possible to use transcriptomic techniques to examine cellular RNA if whole cell analyses are not possible.

- **Humoral Responses**

Humoral immunoglobulin specific levels – including anti-capsule and anti-protein (PspA, PsaA, PspC, Ply) IgG and IgA will be examined by ELISA as well as opsonophagocytic activity (OPA). Some immunoglobulin responses may alter adhesion, or be anti-inflammatory. We will collaborate with CDC Atlanta to optimise Ig response measurement using Luminex (hence parsimonious use of sample) and then develop both adhesion and avidity assays to determine immunoglobulin function.

- **Cellular Responses**

Differential cell counts will be performed by cytospin on all nasal washes and BAL samples, before and after pneumococcal carriage to confirm the absence of inflammation. Antigen specific CD4 T cell responses are critical in defence against pneumococcal carriage [2] in animal models. We will determine these responses in nasal wash if possible (above) and BAL samples using multi-parameter flow cytometry [4]. In particular, we will determine the CD4 Th1/Th2 ratio and gate on memory CD4 T cells to examine the percentage of antigen specific cells against cell free supernatant from the pneumococcus culture broth. A proliferation index (CFSE staining), activation (CD154 signal) and cytokine profile (IFN gamma, IL-4, TNF and IL-2) of the specific cells can be determined to interrogate the functional "quality" of the responsive cells including regulatory T cell responses. We will compare volunteers' baseline and post-carriage observations to determine response to carriage. We will then compare volunteers with carriage to non-carriage subjects, to determine the type of T cell response that is associated with eradication of carriage. Cellular defence (T cell proliferation by CFSE,

intracellular cytokine staining following antigen stimulation) will be measured in peripheral blood (and BAL if available).

- **Bacterial carriage and microbiota evaluation**

Molecular techniques will be employed to determine the carriage density (primers including *lytA*) of pneumococcus and other nasopharyngeal and lower airway microbiota. Modern techniques include 16SrDNA typing, deep sequencing with 454 technology and transcriptomic profiling (in collaboration with Liverpool University, Imperial College, WT Sanger Institute, U Penn or Univ Nijmegen). None of these methods are clinically validated and so we will use conventional techniques to determine the primary endpoint. We will also characterise the dynamics of co-colonisation, particularly of *S.pneumoniae* with staphylococci and *Haemophilus spp (*in collaboration with Emory University).

6. Safety notes

Experimental Human Pneumococcal Challenge

Pneumococcus is responsible for infections including otitis media (OM), sinusitis, pneumonia, bacteraemia and meningitis. The milder forms of infection (OM, sinusitis) are many times more common than the serious invasive forms of disease. While the risk to individuals of developing any infection is very low (10% adults experience colonisation at any time, and the incidence of invasive disease is 20/100 000 patient years), the study is designed to ensure any risk is minimal by appropriate -

- study design
- careful serotype selection and dosing
- volunteer selection and exclusion criteria
- volunteer education and availability of antibiotics
- rigorous safety procedures including daily monitoring
- 24 hour emergency telephone contact with researchers (including close individual daily monitoring) and access to hospital facilities and prompt treatment if required.

We have now experience of inoculating and following healthy volunteers using several serotypes, and a range of doses. Volunteers have experienced no carriage, natural carriage and experimental carriage.

The Data Monitoring and Safety Committee (DMSC) is charged with monitoring the study and advising the PI and study team. It consists of:

(1) Professor Robert C Read, Chair of Infectious Diseases, University of Sheffield (Chair) – supervises Neisseria human inoculation studies and is an expert on mucosal defence against infection
(2) Professor David Lalloo, LSTM – an experienced clinical trialist with substantial experience of DMSC work
(3) Dr Brian Faragher, Senior Lecturer in Statistics, LSTM – is experienced in clinical trials and DMSC work.

The DMSC will receive a weekly update on all recruitment (by email) and will meet by telephone conference in the event of any adverse events. A face to face meeting will be arranged after the dose-ranging studies to make recommendations.

Bronchoscopy and BAL

We have experience of over 1000 of these procedures and have published an audit of volunteer experience in the Journal of Medical Ethics. We have a full risk assessment that can be provided as separate documents but in summary we have had a low complication rate (mild symptoms in less than 25% subjects) and no serious adverse events.

7. Analysis plan

The primary endpoint of carriage will be plotted as a rate against dose and a dose-carriage plot used to predict 50% and 80% carriage (if reached). Using a target carriage rate, LQAS methods will be used to test the confidence limits of the protocol in a reproducibility study.

Immunological parameters will all be compared against pre-exposure values where possible in paired analyses using parametric or non-parametric tests as appropriate. In the case of BAL data, we will only have one sample and therefore and will test hypotheses against test values and by non-paired comparison between groups (e.g. carriers/non-carriers). For example, in current studies we know that the percentage of antigen-specific T cells does not exceed 1 in normal subjects in the absence of carriage.

Hypotheses regarding bacterial co-colonisation will be tested using molecular techniques. These methods are inherently semi-quantitative and so evaluation will be by comparison of proportions in samples at different time points and between subject groups. We will be advised by Dr Brian Faragher and Professor Joe Valadez in the complex analyses planned in this study.

8. Future plan and implications of the work

Success in this project will result in:

a) A robust EHPC protocol that can be used in novel vaccine evaluation.
b) New information regarding mucosal responses and bacterial colonisation with direct application to mucosal vaccine development.

Future work will be planned to build on both of these anticipated outcomes by engaging with vaccine manufacturing companies and mucosal adjuvant programmes.

References

1. Levine, O.S., et al., Pneumococcal vaccination in developing countries. Lancet, 2006. 367(9526): p. 1880-2.

2. Shapiro, E.D., et al., The protective efficacy of polyvalent pneumococcal polysaccharide vaccine. N Engl J Med, 1991. 325(21): p. 1453-60.

3. Obaro, S.K. and S.A. Madhi, Bacterial pneumonia vaccines and childhood pneumonia: are we winning, refining, or redefining? Lancet Infect Dis, 2006. 6(3): p. 150-61.

4. Klugman, K.P., et al., A trial of a 9-valent pneumococcal conjugate vaccine in children with and those without HIV infection. N Engl J Med, 2003. 349(14): p. 1341-8.

5. Holmgren, J. and C. Czerkinsky, Mucosal immunity and vaccines. Nat Med, 2005. 11(4 Suppl): p. S45-53.

6. McCool, T.L., et al., The immune response to pneumococcal proteins during experimental human carriage. J Exp Med, 2002. 195(3): p. 359-65.

7. McCool, T.L., et al., Serum immunoglobulin G response to candidate vaccine antigens during experimental human pneumococcal colonization. Infect Immun, 2003. 71(10): p. 5724-32.

8. Alexander, J.E., et al., Immunization of mice with pneumolysin toxoid confers a significant degree of protection against at least nine serotypes of Streptococcus pneumoniae. Infect Immun, 1994. 62(12): p. 5683-8.

9. Briles, D.E., et al., Intranasal immunization of mice with a mixture of the pneumococcal proteins PsaA and PspA is highly protective against nasopharyngeal carriage of Streptococcus pneumoniae. Infect Immun, 2000. 68(2): p. 796-800.

10. Arulanandam, B.P., et al., Intranasal vaccination with pneumococcal surface protein A and interleukin-12 augments antibody-mediated opsonization and protective immunity against Streptococcus pneumoniae infection. Infect Immun, 2001. 69(11): p. 6718-24.

11. Briles, D.E., et al., Immunizations with pneumococcal surface protein A and pneumolysin are protective against pneumonia in a murine model of pulmonary infection with Streptococcus pneumoniae. J Infect Dis, 2003. 188(3): p. 339-48.

12. Ogunniyi, A.D., et al., Immunization of mice with combinations of pneumococcal virulence proteins elicits enhanced protection against challenge with Streptococcus pneumoniae. Infect Immun, 2000. 68(5): p. 3028-33.

13. McCool, T.L. and J.N. Weiser, Limited role of antibody in clearance of Streptococcus pneumoniae in a murine model of colonization. Infect Immun, 2004. 72(10): p. 5807-13.

14. Richards L, Ferreira DM, Miyaji EN, Andrew PW, Kadioglu A. The immunising effect of pneumococcal nasopharyngeal colonisation; protection against future colonisation and fatal invasive disease. Immunobiology. 2010 Apr;215(4):251-63.

15. Basset A, Thompson CM, Hollingshead SK, Briles DE, Ades EW, Lipsitch M, et al. Antibody-independent, CD4+ T-cell-dependent protection against pneumococcal colonization elicited by intranasal immunization with purified pneumococcal proteins. Infect Immun. 2007 Nov;75(11):5460-4.

16. Tam JC, Grant NL, Freire-Moran L, Tolmie H, French N, Gordon SB. Opsonic function of bronchoalveolar lavage IgG after pneumococcal vaccination in HIV-infected and uninfected adults. J Allergy Clin Immunol. 2009 Jun;123(6):1420-1.

17. Gordon SB, Malamba R, Mthunthama N, Jarman ER, Jambo K, Jere K, et al. Inhaled delivery of 23-valent pneumococcal polysaccharide vaccine does not result in enhanced pulmonary mucosal immunoglobulin responses. Vaccine. 2008 Oct 3;26(42):5400-6.

18. Gordon SB, Kayhty H, Molyneux ME, Haikala R, Nurkka A, Musaya J, et al. Pneumococcal conjugate vaccine is immunogenic in lung fluid of HIV-infected and immunocompetent adults. J Allergy Clin Immunol. 2007 Jul;120(1):208-10.

19. Pagano M, Valadez JJ. Commentary: Understanding practical lot quality assurance sampling. Int J Epidemiol. 2010 Feb;39(1):69-71.
