# Supplementary material for: Pneumococcal colonisation is an asymptomatic event in healthy adults using an experimental human colonisation model
Source: PLoS One. 2020 Mar 10;15(3):e0229558. doi: 10.1371/journal.pone.0229558 (PMC7064211; doi:10.1371/journal.pone.0229558)
Supplement: S3 Fig — (DOCX) [file pone.0229558.s003.docx]

**Study Flowchart**

## Allocation

## Analysis

## Follow Up

Analysed (n=17)
♦ Excluded from analysis (n= 0)

Analysed (n= 8)
♦ Excluded from analysis (n=0)

Assessed for eligibility (n= 55)

**SPN 6B**

Allocated to intervention (n=29)

♦ Received allocated intervention (n= 29)

Lost to follow-up (n= 0)

Discontinued intervention (n=0)

Lost to follow-up (n= 0)

Discontinued intervention (n=0)

Lost to follow-up (n=0)

Discontinued intervention (n= 0)

**Saline**

Allocated to intervention (n= 8)

♦ Received allocated intervention (n= 8)

**SPN 23F**

Allocated to intervention (n=17)

♦ Received allocated intervention (n=17)

Excluded (n= 1)

♦  Not meeting inclusion criteria (n=0 )

♦  Declined to participate (n= 1)

♦  Other reasons (n= 0)

Analysed (n= 29)
♦ Excluded from analysis (n=0)
