# Supplementary material for: Pneumococcal colonisation is an asymptomatic event in healthy adults using an experimental human colonisation model
Source: PLoS One. 2020 Mar 10;15(3):e0229558. doi: 10.1371/journal.pone.0229558 (PMC7064211; doi:10.1371/journal.pone.0229558)
Supplement: Attachment — Submitted filename: Response to reviewers.docx [file pone.0229558.s004.docx]

Dr Andrea Collins

Senior Clinical Lecturer

Honorary Respiratory Consultant Royal Liverpool University Hospital

and University Hospital Aintree

Liverpool School of Tropical Medicine

Pembroke Place

Liverpool

L3 5QA

[Andrea.collins@LSTMed.ac.uk](mailto:Andrea.collins@LSTMed.ac.uk)

18/09/19

Dear PLOS ONE

RE: **Pneumococcal Colonisation is an Asymptomatic Event in Healthy Adults using an Experimental Human Colonisation Model**

This manuscript was previously submitted to PLOS ONE in 2016 (PONE-D-16-30234). At that time it was sent for peer review but unfortunately the maximum time for response elapsed before the manuscript could be re-submitted. We would like to now re-submit this manuscript for consideration of publication.

It has now been re-written and formatted and has new analysis and comment. We are grateful for the helpful feedback from the peer reviewers and have addressed each detailed point below. The responses are highlighted in bold.

**Review Comments to the Author**

Reviewer #1: The authors have presented a clearly explained analysis of a small trial (N=55) of experimental inoculation with serotype restricted pneumococcus (ie 6B or 23F) or saline, with the latter being used as a control. I believe that this study should be published with a few minor modifications which I'll suggest below referring to the kindly provided line numbers as required. Most of the comments raised are somewhat matters of style/opinion which the authors may chose to ignore but I would like them to consider. However there is one place in a table where I think there is an error, that maybe typographical, but certainly requires revision.

1) Lines 70-71: I personally find the term "trend towards" to be somewhat misleading in reference to interpreting P value and much prefer the nomenclature of Kirkwood and Sterne who use the term "weak evidence" (Essential Medical Statistics 2nd Ed p75). However, I do realize that this is somewhat matter of taste.

**Thanks, this has now been removed following a re-write of the manuscript.**

2) Line 110: I believe there is a missing "been" required between "has" and "noted"

**Thanks, this has now been removed following a re-write of the manuscript.**

3) Line 146: Shouldn't "your" be "our"?

**Thanks you, this has now been removed following a re-write of the manuscript.**

4) Lines 218 and 255-6: The issue of "trend towards"

**This has now been removed following a re-write of the manuscript, thanks.**

5) Figs 2 and 3 : The authors might want to consider changing the Y axes by curtailing the maximum to 4 on the symptom score. I appreciate that the max value of 7 must be derived from the scoring system but I think being able to better distinguish the possible comparisons by spreading apart the lines (particularly in Figure 3) may be worth it.

**Thanks for pointing this out, the y- axis on the figures has been altered to make this clearer.**

6) Table 1: The percentage male is wrong at 50% and should be corrected to 57% ie 4/7

**Agreed, Table 1 has been removed as the data is now presented in a series of Figures, thanks.**

Reviewer #2: The authors describe some further data from their successful and previously well-described Experimental Human Pneumococcal Challenge (EHPC) model. This manuscript aims to look specifically at the rate of symptom reporting in healthy adult individuals taking part in the study, who were challenged with 2 different serotypes of pneumococcus or saline control.

1) Overall, the quality of the writing is low, such that in places the sentences are not understandable to the informed reader (for example, lines 145-148; line 151 "We published our protocol with full safety cover"). This is especially noticeable in the methods, where previous publications and methodologies are referred to, without any further description or detail given here. This makes it not possible to understand the procedures and rationale without search for the earlier work after every sentence. I think that this is especially important in human challenge studies, the manuscripts from which will be held to a higher standard due to the perceived risk of the work involved.

**The paper has now been re-written to take this feedback into account, thank you. The methods section has been replaced to specifically address this.**

2) The data given in the results (where comprehensible - see lines 218-220), and in particular Table 2, are insufficiently described and analysed to draw the conclusions made by the authors. For example, there is no statistical evidence of a trend provided, not are the symptoms grouped in such a way as to be able to spot this. Furthermore, the table only gives symptoms of severity >=3; presumably there were milder symptoms reported also, scored 1 or 2 but both under the category 'none' which is unclear to me.

**The data has now been presented in a clearer manner. Table 2 has been removed.**

**The rates of symptoms reported between groups have now been compared using Fisher’s exact tests and Chi square where it was deemed appropriate. A Spearman’s rank has been used to assess for evidence of correlation.**

**The symptoms were assessed using a clear symptom log. In order to address any issues of clarity regarding this it has been made more well defined in Figure 1.**

**‘Asymptomatic’ was defined as any symptom with a score graded < 2. It was felt that symptoms regarded by the participant as ‘none to occasional limited episode’ were not *clinically* significant.**

3) All of the figures require review, specifically with respect to the scale on the y-axes. In addition, figure 3 presents categorical data with connecting lines between different symptom types, which I think is not appropriate. Please also note that the numbering of the Figures is incorrect.

**Thanks, the Y-axis of Figures has been altered to improve their clarity. The number of Figures in the text has been updated in the new manuscript.**

While I think there may be data here that are worth reporting, the data provided and the analyses performed do not allow the reviewer to make that assessment.

Reviewer #3: The manuscript entitled “Pneumococcal Colonisation is an Asymptomatic Event in Healthy Adults using an Experimental Human Colonisation Model” compares the symptoms of human volunteers colonized, or not, with pneumococcus -S. pneumoniae-) upon intranasal challenge. The authors conclude that in adults, colonisation with pneumococcus does not induce symptoms. Similarly, co-colonisation with viruses results no symptoms. These results differ from in children, who develop various symptoms associated with the upper respiratory tract, as demonstrated in previously published publications.

This is a follow-up manuscript from this research group, focusing only in the symptoms from the volunteers. The current manuscript is well written, simple and provides a single valid conclusion. However, before publication, the authors need to fix various parts of the text and figures.

Minor comments:
1) Harmonize the text with the symptoms. In some parts asymptomatic are described as <= 3 (e.g., line 173) while in the Figure 2 it is indicated <= 2.

**Thank you, this has been clarified throughout the text. Asymptomatic has been defined as a score < 2.**

2) Legends for Figure 2, 3 and 4 are mislabelled (Pages 18-20)

**Thanks, Figures 2,3 and 4 have been re-labelled.**

3) Figure 4a. Score of nasal symptoms. Y Axis difficult to read. Either change font or write the text in 2 lines

**The axis of Figure 4 has been adjusted and the graph has been made clearer, thank you.**

4) Study participants were infected with 2 pneumococcal strains 6B and 23F. Only 6B colonised the participants. Please include in the discussion section the reasons the authors believe that the strain 23F did not colonise the volunteers.

**The reason for the difference in colonisation between the two SPN strains is that different isolates of different serotypes colonise at different rates in different hosts in different conditions. We feel that this is however beyond the scope of this study which instead focuses on whether colonisation itself is a symptomatic event.**

5) The Y axis in figures 2-4 go from 1 to 7, due to the symptom score used. However, the higher score recorded was around 3. I would suggest splitting the Y axis to display the data better, particularly in figure 3 (correlation).

**Thanks, the y-axis has been altered to improve the clarity of the graph.**

Yours sincerely,

Dr Andrea Collins and Prof Daniela Ferreira
