# Supplementary material for: Pneumococcal colonisation is an asymptomatic event in healthy adults using an experimental human colonisation model
Source: PLoS One. 2020 Mar 10;15(3):e0229558. doi: 10.1371/journal.pone.0229558 (PMC7064211; doi:10.1371/journal.pone.0229558)
Supplement: Attachment — Submitted filename: PLOS one symptoms rebuttal letter 16.12.19.docx [file pone.0229558.s005.docx]

Dr Andrea Collins

Senior Clinical Lecturer

Honorary Respiratory Consultant Royal Liverpool University Hospital

and University Hospital Aintree

Liverpool School of Tropical Medicine

Pembroke Place

Liverpool

L3 5QA

[Andrea.collins@LSTMed.ac.uk](mailto:Andrea.collins@LSTMed.ac.uk)

16/12/19

Dear PLOS ONE

RE: **Pneumococcal Colonisation is an Asymptomatic Event in Healthy Adults using an Experimental Human Colonisation Model**

Thank you for your peer review comments dated the 01/12/19. We are grateful for the helpful feedback and have addressed each detailed point below. The responses are highlighted in bold.

**Review Comments to the Author**

Reviewer #1:

The manuscript by Trimble and colleagues present additional results from an experimental human challenge model, describing whether experimental pneumococcal colonisation in healthy adults is a symptomatic event. This manuscript is a resubmission from 2016 (but due to time limits is now a new submission) which has addressed some of the queries from the initial review. This study reports some interesting data but is nevertheless limited mainly due to the small sample size, especially for the secondary analyses.

Some points for consideration:

1. The pneumococcal colonisation rate was 30.4% (14/46), which was solely due to serotype 6B, and actually 48% if only those who were inoculated with 6B are included. This seems quite high for an otherwise healthy adult population. The authors state that this model mimics natural pneumococcal exposure but the high carriage rate may suggest otherwise. Can the authors provide some comment on the colonisation rate they observed with respect to what has been reported among UK healthy adults or more generally? Some explanation of this would greatly benefit the manuscript. The authors also argued against providing some comment on the lack of 23F colonisation but I tend to think that this would also be worth noting in the discussion, given that both these are commonly carried serotypes.

**Thank you for your feedback, the EHPC model is able to artificially induce the otherwise naturally occurring phenomenon of pneumococcal carriage by pipetting the live bacteria directly into the participant’s nasopharynx. As the EHPC inoculation is more efficient than the ‘natural process’ the rate of colonisation is higher. This has now been added to the discussion. We have added an explanation for the difference in colonisation rate between serotypes as requested.**

2. Figures 2 and 3 could be combined since they are both essentially reporting the same data but with a different comparison. Also, it should be Fisher’s exact test, not Fischer’s exact test.

**Thank you, we have now corrected the spelling of Fisher’s exact test. We feel that combining the two figures together may affect the clarity of the underlying message, and therefore have respectfully chosen to keep them separate. We have however added a legend to all figures to make them easier to interpret.**

3. Can the authors comment on the apparent high rate of nasal and non-nasal symptoms in the control group and whether they believe this could have contributed to the lack of any clinical symptoms being demonstrated for the other groups? An independent assessor may have been useful here.

**We recognise that there is a high number of symptoms in the control group. We believe this may be due in part to the small study sample size but also reflects the high sensitivity of the Likert scale used to record any symptoms. Since the symptoms were self-reported by participants and they were blinded to their intervention group, we feel that an independent assessor would have been unlikely to improve this.**

4. There are a couple of instances where what is reported in the results is inconsistent with the data or discussion. For example, on lines 192-193 it states that “This study does not however report the effect of co-colonisation (SPN and virus) on symptoms” when this is clearly shown in Figure 5. Also on lines 207-208, it states that “…experimental SPN colonisation does not increase nasal or non-nasal symptoms” when in the results (lines 156-157) it states that “Experimental SPN colonisation rates were higher in the presence of virus….(p<0.05)”. Please correct. The p-value, if indeed significant, should be included in the Figure.

**Thank you, the comment regarding symptoms from co-colonisation refers to the Rodrigues at al study. This has been made clearer in the text to avoid confusion.**

**In the discussion we note that experimental SPN colonisation does not increase nasal or non-nasal symptoms. In the results section we note that experimental SPN colonisation was higher in the presence of virus. This is referring to two separate points. Participants who are positive for the presence of a virus and SPN (‘co-colonised’) did not appear to have greater rates of symptoms. The figure relating to this data (Figure 5) has been edited to include the relevant p- values and a legend has been added for clarity.**

5. What were the viruses that were detected, and were there any associations between specific viruses?

**A variety of viruses were detected, however since these were only present in very small numbers, we were unable to ascertain any relationship between symptoms and these specific viruses. In future studies we aim to further investigate this relationship in more detail.**

For most of the reported p-values there are no = sign. Please include.

**Thank you, this has now been added.**

Reviewer #2: General comments

This study uses a human challenge model to examine if symptoms are experienced in the context of pneumococcal colonisation among adults. The structure of the paper is acceptable, however there could be improvement made in the finer details, e.g. error bars/confidence intervals and raw numbers

I think it is also important to mention the limitations of this study e.g. not an RCT, low sample size, and only one serotype successfully colonised. Consequently I think some of the statements in the discussion should have the language softened, as this study is not the definitive study which demonstrates that colonisation is asymptomatic among adults. Rather it might suggest this, but further work using more serotypes, in larger RCTs is needed.

**Thank you, we have modified the manuscript to take this into account. Changes have been made to the abstract, discussion and conclusion to recognise this.**

Specific comments

Abstract

Methods section line 46

" ... reported between groups was compared ..." to groups were compared

**Thank you, this has now been updated.**

Results section line 49

S. pneumoniae (29 with serotype 6B, 17 with serotype 23F ...

**Thanks, this has been updated**

Conclusion section line 58

Pneumococcal colonisation using the EHPC was asymptomatic ...

**Thanks, this has also been updated as requested.**

Introduction

line 69 - correct typo, H. influenzae

**Thanks, this has now been updated**

line 70-73 - could be better worded

**This has been re-worded for clarity.**

lines 83-87 - suggest rearranging this paragraph e.g.

The experimental pneumococcal challenge model (EHPC) mimics natural colonisation in healthy ....vaccines in randomised control trials. We aimed to use the EHPC to investigate if the process is symptomatic....

**Thank you this has now been updated.**

Methods

Was a sample size calculation conducted?

**Thank you for this feedback. We have not performed a sample size calculation as this was performed as a pilot study. In a larger follow up study we will ensure that a formal sample size calculation is done.**

Results

line 134-137

please provide actual numbers, proportions, confidence intervals, and p-values.

**These have been added where required.**

figures - please add in confidence intervals.

**Thank you, these have been included where required. A legend has been added to aid the interpretation.**

Discussion

Lines 164 and 212

I would tone down the language here e.g.

This study provides evidence supporting the hypothesis that SPN colonisation among adults is asymptomatic

**Thank you, this has been updated. We have taken this into account and altered the discussion and conclusion to reflect this.**

line 171

Other than sample size, key limitations also include: the study wasn't randomised, only one serotype was assessed in this study, and pre-existing immunity.

**The lack of randomisation to the allocated group and the use of a single successful serotype has been added as key limitations. As we used SPN 6B, a serotype that is not otherwise present in the community, it is unlikely that the participants have pre-existing immunity to this serotype. A line has been added to the discussion in order to address this.**

Yours sincerely,

Dr Andrea Collins and Prof Daniela Ferreira
